# Computational Investigations of Traditional Chinese Medicinal Compounds against the Omicron Variant of SARS-CoV-2 to Rescue the Host Immune System

**DOI:** 10.3390/ph15060741

**Published:** 2022-06-13

**Authors:** Ziad Tareq Naman, Salim Kadhim, Zahraa J. K. Al-Isawi, Christopher J. Butch, Ziyad Tariq Muhseen

**Affiliations:** 1 Department of Medical Laboratory Techniques, Al-Ma’Moon University College, Aladhamia, Baghdad 72029, Iraq; mscziad86@gmail.com; 2 College of Pharmacy, University of Alkafeel, Najaf 61001, Iraq; sfk8@leicester.ac.uk; 3 Department of Pharmacology and Toxicology, College of Pharmacy, University of Kufa, Najaf 61001, Iraq; zahraaj.kadhim@uokufa.edu.iq; 4 Department of Biomedical Engineering, College of Engineering and Applied Sciences, Nanjing University, Nanjing 210093, China; 5 State Key Laboratory of Analytical Chemistry for Life Science, Jiangsu Key Laboratory of Artificial Functional Materials, Nanjing University, Nanjing 210093, China; 6 Department of Pharmacy, Al-Mustaqbal University College, Hillah, Babylon 51001, Iraq

**Keywords:** SARS-CoV-2, COVID-19, NSP3, TCM, MD simulations

## Abstract

Macrodomain-I of the NSP3 (non-structural protein 3) is responsible for immune response hijacking in the SARS-CoV-2 infection known as COVID-19. In the omicron variant (B.1.1.529), this domain harbors a new mutation, V1069I, which may increase the binding of ADPr and consequently the infection severity. This macrodomain-I, due to its significant role in infection, is deemed to be an important drug target. Hence, using structural bioinformatics and molecular simulation approaches, we performed a virtual screening of the traditional Chinese medicines (TCM) database for potential anti-viral drugs. The screening of 57,000 compounds yielded the 10 best compounds with docking scores better than the control ADPr. Among the top ten, the best three hits—TCM42798, with a docking score of −13.70 kcal/mol, TCM47007 of −13.25 kcal/mol, and TCM30675 of −12.49 kcal/mol—were chosen as the best hits. Structural dynamic features were explored including stability, compactness, flexibility, and hydrogen bonding, further demonstrating the anti-viral potential of these hits. Using the MM/GBSA approach, the total binding free energy for each complex was reported to be −69.78 kcal/mol, −50.11 kcal/mol, and −47.64 kcal/mol, respectively, which consequently reflect the stronger binding and inhibitory potential of these compounds. These agents might suppress NSP3 directly, allowing the host immune system to recuperate. The current study lays the groundwork for the development of new drugs to combat SARS-CoV-2 and its variants.

## 1. Introduction

Coronavirus disease-2019 (COVID-19) is defined as a disease caused by a novel coronavirus called severe acute respiratory syndrome coronavirus 2 (SARS-CoV-2) [1]. SARS CoV-2 rapidly spread from person to person, and serious human disease was in recent times described in the city of Wuhan, Hubei Province, China. Coronavirus causes headache, fever, and respiratory disease, e.g., cough and shortness of breathing. SARS-CoV-2 infects humans through direct binding to host cell entry proteins (spike). The six functional open reading frames (ORFs) are well-arranged in order from 5′ to 3′ replicas (ORF1a/ORF1b), membrane (M), envelope (E), spike (S), and nucleocapsid (N), whereas nonstructural protein include3-chymotrypsin-like protease, papain-like protease, and RNA-dependent RNA polymerase, is encoded by the ORF region [2,3].

A SARS-CoV-2-associated global pandemic, with unceasing chaos, has now reported many variants of the virus [4]. Among the reported variants, many are particularly associated with immune response evasion, higher transmissibility, increased morbidity, and re-infection. Until now, these variants have been deemed either as variants of interest (VOIs) or variants of concern (VOCs) based on the threat they pose to public health. For instance, the alpha variant, also known as B.1.1.7, with 40–80% increased transmission, includes 69–70 del, N501Y, and P681H mutations, while beta, also called B.1.1.351, gamma, delta, and B.1.1.529 variants are classified as VOCs [5,6]. Moreover, a new version of B.1.1.7 supplemented with the E484K mutation was reported to be associated with 39 confirmed patients [7]. Moreover, B.1.351, which was reported in South Africa and harbors K417N, E484K, and N501Y variations, which were reported to increase the transmission and decrease the T cell-triggered immune response against COVID-19 infection, was recorded. In early 2021, P.1 variant was discovered in Brazil with K417T, E484K, and N501Y mutations in the RBD with transmission increased by 38% and mortality by 50% [8]. A molecular modelling study based on protein coupling deciphered the mechanism of higher infection associated with these variants of SARS-CoV-2 [9]. The binding variations were deeply discovered by demonstrating the interaction pattern and dynamic features of the complexes. In October 2020, India detected a more lethal strain of SARS-CoV-2, officially known as B.1.617.2. This variation has L452R, T478K, and P681R in the RBD, resulting in an 87 percent increase in transmissibility, an 85 percent increase in hospitalizations, and a 137 percent rise in death. Later, in India and the United Kingdom, a novel variant known as “Delta plus” was discovered with an additional mutation, K417N [10]. Similarly, a novel variant termed as Mu or B.1.621 was discovered to have spike protein alterations, some of which are shared with other VOCs. R346K, Y144T, Y145S, and insertion at position 146N are among the new mutations in this variant [11]. L452Q and F490S mutations in RBD are found in a unique VOI known as C.37 or its variant, which lowers antibody-mediated neutralization [12]. The B.1.617.1 strain with the L452R mutation in the (RBD) receptor-binding domain, on the other hand, has been attributed to decreased antibody neutralization by altering the corresponding conformational epitopes. Furthermore, the VOI Iota (lineage B.1.526) carries the mutation E484K, which confers resistance to therapeutic monoclonal antibodies and makes it more resistant to neutralization [13].

The most recent omicron strain, formally known as B.1.1.529, was discovered in November 2019 in South Africa and has 30 mutations in the spike protein including A^67V^, Δ69–70, T^95I^, G^142D^, Δ143–145, Δ211, L^212I^, ins214EPE, G^339D^, S^371L^, S^373P^, S^375F^, K^417N^, N^440K^, G^446S^, S^477N^, T^478K^, E^484A^, Q^493R^, G^496S^, Q^498R^, N^501Y^, Y^505H^, T^547K^, D^614G^, H^655Y^, N^679K^, P^681H^, N^764K^, D^796Y^, N^856K^, Q^954H^, N^969K^, and L^981F^, among which 15 mutations lie in the RBD [14]. Other proteins also reported mutations, such as NSP3 (K^38R^, V^1069I^, Δ1265, L^1266I^, A^1892T^), NSP4 (T^492I^), NSP5 (P^132H^), NSP6 (Δ105–107, A^189V^), and NSP12 (P^323L^), while I^42V^ was reported in NSP14. Furthermore, sub-lineages, i.e., BA.1/B.1.1.529.1, BA.2/B.1.1.529.2, and BA.3/B.1.1.529.3, were reported by WHO (World Health Organization) as a threat to public health [15]. The variant is a major health concern all around the globe, and the therapeutic effectiveness of existing vaccines against it is yet unknown [16,17]. More research is needed to determine the molecular basis of pathogenicity in the omicron variant. Furthermore, new therapies against the recently emerging SARS-CoV-2 strains would require the use of advanced techniques.

The novel coronavirus-2019 genome encodes four structural proteins (S, M, E, and N), in which the S-protein gives the virus its corona-like shape, which is mainly responsible for the attachment to host cell receptors (ACE2s) or surface proteins, and 16 non-structural proteins (NSP1 to NSP16). The binding of spike protein to ACE2 (Angiotensin-Converting Enzyme 2) of the host initiates the infection in cells. The ACE2 is mainly expressed in the lungs, kidney, and small intestine, leading to serious illness [18]. During infection, the host cell protease cleaves the S-protein at the S1/S2 cleavage site. This priming (cleavage of S-protein) results in the division of protein into the S1 ectodomain at the N-terminal and the S2 membrane-anchored domain at the C-terminal. The S1 subunit recognizes the associated cell surface receptor, while the latter assists viral entry [19]. The SARS-CoV S1 subunit of the spike protein has conserved 14 aa in the RBD, which functions to recognize ACE2 and can infect both humans and bats. Among this conserved 14 aa in SARS-CoV, eight residues are highly conserved in 2019-nCoV, supporting the assumption that ACE2 is also the receptor of this new virus [20]. Among these proteins, the main proteins, PLpro, spike, and RdRp, are deemed to be direct drug and vaccine targets, but other proteins are also essential for drug design [21]. For instance, the Mac-I of the largest protein, NSP3, has also been deemed as an attractive drug target because of its role in disrupting the innate immune reaction and increasing of virulent properties of the virus [22]. NSP3 is comprised of three macrodomains and two adjacent SUD-M-like domains that have been reported to be associated with NSP3 functional modulation. Mac-I has a significant role in viral pathogenesis and increases interferons’ responses for viral neutralization [23]. Further reports have also disclosed the hijacking of the host immune response by Mac, which is carried out through interference with the IFN (Interferons) pathway and malfunctioning of STAT1 (signal transducer and activator of transcription 1) [24,25]. This hijacking of the IFN and STAT1 pathways has been reported to be possibly linked with the cytokines storm phenomena [26,27]. 

More research is needed to cope with this alarming pandemic situation in order to create safe and effective treatments swiftly. In this context, the macrodomain is thought to be the most druggable target for the development of COVID-19 therapy [28,29]. As a result, we used computational molecular screening to investigate the binding affinity of drugs against the macrodomain-I of the omicron (B.1.1.529) variant. The current study employs molecular docking and simulation-based methods [30,31,32] to identify potential anti-viral compounds from the TCM database against SARS-CoV-2. The findings provide crucial information on the antiviral effectiveness of the evaluated drugs against SARS-CoV-2. The findings will aid in the development and identification of potential medicinal solutions for the treatment of COVID-19.

## 2. Results and Discussion

### 2.1. Macrodomain of Omicron (B.1.1.529) and Structural Modelling

**Figure 1:**
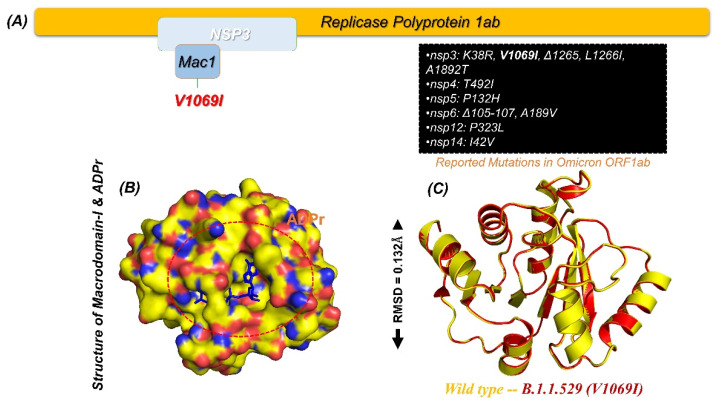
(**A**) NSP3 and reported mutations in the omicron variant. (**B**) Surface representation of Mac-I in complex with ADPr. (**C**) Superimposed wild type a V1069I mutation in Mac-I.

The continuously emerging variants of SARS-CoV-2 are a greater threat to public health [33]. These variants are associated with high infection, transmission, and re-infection. Moreover, some of the previous variants such as B.1.1.7, B.1.617, P.1, and A.30 have also been reported to escape the immune response. Recently, B.1.1.529, or the omicron variant, first identified in South Africa with the highest number of mutations ever, has been reported to be associated with higher transmissibility than other variants. All the proteins encoded by the genome of SARS-CoV-2 are responsible for essential roles in virulence and replication in different stages of infection [34,35,36]. Among these proteins, the largest and multi-domain protein called non-structural protein 3 or NSP3 is an essential factor for efficient translational and replication processes. NSP3 has eight functional domains that also include macrodomains, which play significant roles in viral escaping when attacked by the innate immune response. Among the macrodomains, macrodomain-I (X-domain) in particular also shares high sequence and structural conservation. Mac-I binds ADPr, and the strength of binding is directly associated with the pathogenicity level [37]. For instance, binding strength reduction or the complete loss of ADPr binding has been reported to be related to less or no infection in the IBV model [38]. Any mutation in the structure is associated with functional variations induced by conformation changes in the protein structures. For instance, K38R, V1069I, L1266I, and A1892T, as well as a deletion (Δ1266I) have recently been reported in the NSP3 protein of the omicron variant, as shown in Figure 1A. The Mac-I in the NSP3 starts from 1025–1194, and a single V1096I has been reported in this region, which may increase the binding of ADPr and consequently increase the pathogenesis by hijacking the innate immune response given in Figure 1A. Mac-I is also a potential therapeutic target; therefore, finding small molecule inhibitors for this domain might aid in the restoration of the host immunological innate response. For instance, a carbazole-based drug known as GeA-69 has been previously tested to target the human macrodomain to treat various types of cancers [39]. Similarly, structure-based approaches are being used to find drugs that target viral macrodomains [40]. New powerful therapies are needed to block Mac-I involvement in SARS-CoV-2 pathogenesis. Hence, our research combines structure-based drug discovery with molecular modelling to find new compounds that effectively target the Mac-I and salvage the innate response. The wild-type structure was used to construct a V1069I mutant of the Mac-I from the omicron variant (Figure 1B). The modelled V1069I-Mac-I, as shown in Figure 1C, demonstrated an RMSD (root mean square deviation) difference of 0.132 Å. The minimal RMSD difference demonstrated the accurate folding and distribution of secondary structural elements. 

### 2.2. Virtual Screening and Re-Docking of TCM

Using EasyVS, an online web tool, the entire TCM database was screened against the Mac-I of the omicron variant. Prior to screening, the RO5 filter was applied, which reported 20,124 drugs that violated Lipinski’s rule of five. Among the total of 57,000 compounds, 36,876 compounds were screened against the binding site of ADPr. Among these, 14,255 compounds reported a docking score less than −4.0 kcal/mol, and 16,453 compounds reported a docking score less than −8.0 kcal/mol. Among the remaining compounds, 5221 compounds reported a docking score of less than −9.0 kcal/mol. Among the remaining 947 compounds, only 356 were reported to have docking scores greater than −9.5 kcal/mol. Compounds with docking scores greater than −9.50 kcal/mol were considered as the threshold, because for ADPr the docking score was previously reported to be −9.46 kcal/mol. The top scoring 50 compounds were selected for re-docking using AutoDock Vina. Among these 50 compounds, 10 compounds reported the best docking score and are given in Table 1. Among the top 10 compounds, TCM42798 had a docking score of −13.70 kcal/mol, TCM47007 of −13.25 kcal/mol, TCM30675 of −12.49 kcal/mol, TCM27763 of −11.93 kcal/mol, TCM33425 of −11.72 kcal/mol, TCM28788 of −11.46 kcal/mol, TCM42159 of −11.45 kcal/mol, TCM47184 of −11.36 kcal/mol, TCM31603 of −11.04 kcal/mol, and TCM31784 of −11.02 kcal/mol. Among the top 10 compounds reported to have higher docking scores than ADPr, only the top three were selected for further analysis, such as interactions, simulation, and post-simulation analysis. In addition to the top three compounds, TCM27763 and apigenin-bioside have been previously reported to have anti-adenovirus activity, which confirms the anti-viral activity of these compounds [41]. It was reported that apigenin reduces adenovirus replication and associated cellular toxicity. For instance, TCM28788 and TCM42159 have been reported to have anti-dengue viral properties, which demonstrate the anti-viral potential of these molecules [42]. The anti-herpes simplex virus type 2 infection activity of tibeticanol and the other compounds are well documented, and it has been reported that these compounds halt viral replication [43]. 

**Figure 2:**
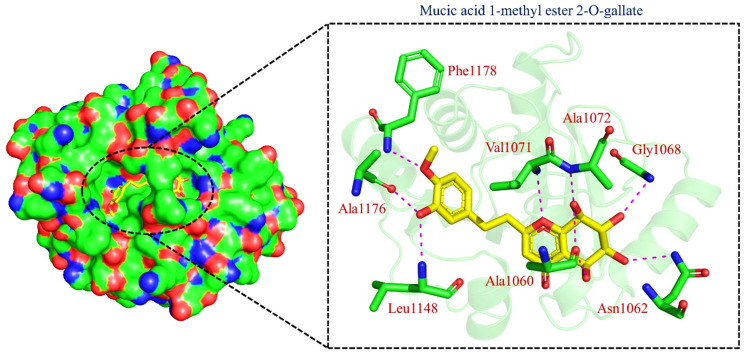
Binding of TCM42798 in the cavity of Mac-I. The right surface representation shows the binding mode of *mucic acid 1-methyl ester 2-O-gallate* (yellow sticks) inside the binding cavity. The right panel represent the 3D interaction pattern, where the TCM42798 is shown as yellow sticks, while the interacting residues are given in green sticks. The interactions (hydrogen bonds) are shown in pink color.

TCM42798, or *mucic acid 1-methyl ester 2-O-gallate*, was reported to have the best docking score of −13.70 kcal/mol. Mucic acid 1-methyl ester 2-O-gallate established a total of nine hydrogen bonds with Ala1060, Asn1062, Gly1068, Val1071, Ala1072, Leu1148, Ala1176, and Phe1178. For instance, these residues were previously reported to have an important role in the binding of ADPr. Comparatively, the docking score for ADPr has been reported to be −9.46 kcal/mol [22]. This shows the strongest binding of TCM42798 and consequently produces an inhibitory effect on Mac-I. The binding pattern of TCM42798 is given in Figure 2.

**Figure 3:**
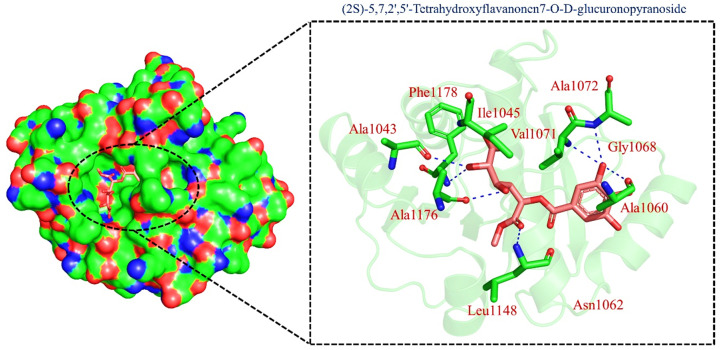
Binding of TCM47007 in the cavity of Mac-I. The right surface representation shows the binding mode of TCM47007 or *(2S)-5,7,2’,5’-Tetrahydroxyflavanone 7-O--D-glucuronopyranoside* (orange sticks) inside the binding cavity. The right panel represents the 3D interaction pattern, where the TCM47007 or *(2S)-5,7,2’,5’-Tetrahydroxyflavanone 7-O--D-glucuronopyranoside* is shown as orange sticks, while the interacting residues are given in green sticks. The interactions (hydrogen bonds) are shown in blue color.

**Figure 4:**
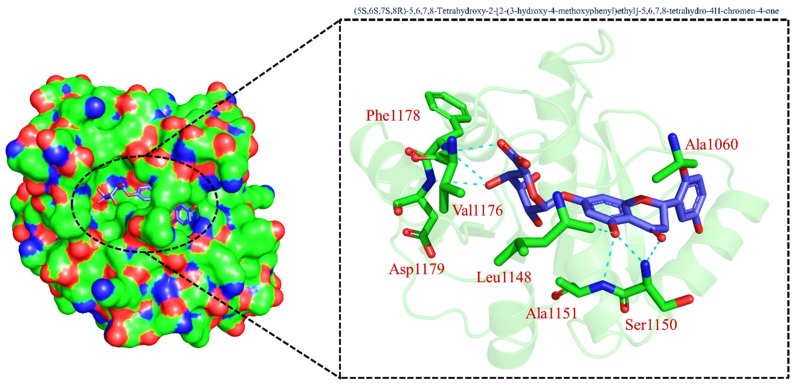
Binding of TCM30675 or *(5S,6S,7S,8R)-5,6,7,8-Tetrahydroxy-2-[2-(3-hydroxy-4-methoxyphenyl)ethyl]-5,6,7,8-tetrahydro-4H-chromen-4-1* in the cavity of Mac-I. The right surface representation shows the binding mode of TCM30675 or *(5S,6S,7S,8R)-5,6,7,8-Tetrahydroxy-2-[2-(3-hydroxy-4-methoxyphenyl)ethyl]-5,6,7,8-tetrahydro-4H-chromen-4-1* (blue sticks) inside the binding cavity. The right panel represent the 3D interaction pattern, where the TCM30675 or *(5S,6S,7S,8R)-5,6,7,8-Tetrahydroxy-2-[2-(3-hydroxy-4-methoxyphenyl)ethyl]-5,6,7,8-tetrahydro-4H-chromen-4-1* is shown as orange sticks, while the interacting residues are given in green sticks. The interactions (hydrogen bonds) are shown in blue color.

On the other hand, TCM47007, or *(2S)-5,7,2’,5’-Tetrahydroxyflavanone 7-O--D-glucuronopyranoside*, also demonstrated a good interaction profile by predicting the docking score of −13.25 kcal/mol. Unlike TCM42798, this complex reported only seven hydrogen bonds with the key residues required for interaction with ADPr. As shown in Figure 3, Ala1043, Ala1060, Gly1068, Val1071, Ala1072, and Ala1176 are involved in hydrogen bonding interactions. This shows the strongest binding of TCM47007 and consequently produces an inhibitory effect on Mac-I. Moreover, the interaction pattern for TCM30675, or *(5S,6S,7S,8R)-5,6,7,8-Tetrahydroxy-2-[2-(3-hydroxy-4-methoxyphenyl)ethyl]-5,6,7,8-tetrahydro-4H-chromen-4-1*, was also analyzed to demonstrate the binding mode of TCM30675. Residues such as Ala1060, Val1176, Phe1178, and Asp1179 are involved in direct interactions with the Mac-I. This shows the strongest binding of TCM30675 and consequently produces an inhibitory effect on Mac-I. The binding pattern of TCM42798 is given in Figure 4.

### 2.3. Dynamic Stability Analysis of the Top Compounds

**Figure 5:**
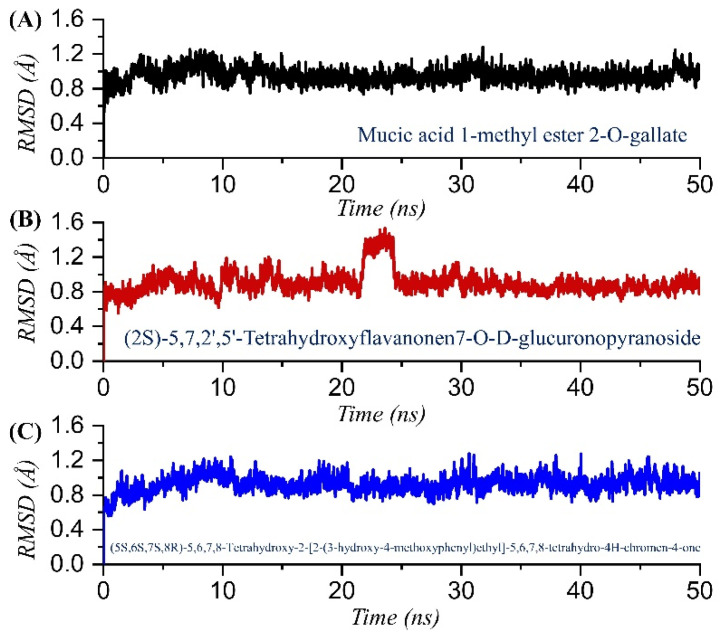
Dynamic stability analysis of the TCM42798, TCM47007, and TCM30675 complexes. (**A**) The RMSD graph for mucic acid 1-methyl ester 2-O-gallate. (**B**) The RMSD graph for TCM47007 or (2S)-5,7,2’,5’-Tetrahydroxyflavanone 7-O--D-glucuronopyranoside. (**C**) The RMSD graph for TCM30675 or (5S,6S,7S,8R)-5,6,7,8-Tetrahydroxy-2-[2-(3-hydroxy-4-methoxyphenyl)ethyl]-5,6,7,8-tetrahydro-4H-chromen-4-1 complex.

Analysis of the drug-bound protein complexes to decipher the structural stability is a key process in determining the inhibitory potential of the interacting compound. To foresee the dynamic stability of each top-scoring compound, we calculated the root mean square deviation (RMSD) for all of the simulation trajectories. As given in Figure 5A–C, all the complexes attained equilibrium at the earlier simulation and reached stability at 1.0 Å. In the case of *mucic acid 1-methyl ester 2-O-gallate*, the complex demonstrated overall stable dynamics with no significant deviation, except at the start of the simulation (1–10 ns) when the structure reported minor acceptable deviation and then reached the equilibrium point with a uniform RMSD graph. The average RMSD reported for the *mucic acid 1-methyl ester 2-O-gallate* complex was reported to be 1.0 Å. The RMSD for *mucic acid 1-methyl ester 2-O-gallate* is given in Figure 5A. The RMSD for TCM47007 or *(2S)-5,7,2’,5’-Tetrahydroxyflavanone 7-O--D-glucuronopyranoside* reported comparatively small unstable dynamics until 15 ns, but then RMSD stabilized and attained equilibrium. As given in Figure 5B, after 15 ns the structure attained stability and demonstrated an average RMSD of 1.1 Å. Furthermore, the RMSD of TCM30675 or *(5S,6S,7S,8R)-5,6,7,8-Tetrahydroxy-2-[2-(3-hydroxy-4-methoxyphenyl)ethyl]-5,6,7,8-tetrahydro-4H-chromen-4-1* demonstrated more similar behavior to the *mucic acid 1-methyl ester 2-O-gallate* complex. The RMSD initially increased until 10 ns and then attained equilibrium. The complex reported no significant deviation except a small deviation between 10 and 20 ns, and then it stabilized again. An average RMSD of 1.2 Å was reported for TCM30675 or *(5S,6S,7S,8R)-5,6,7,8-Tetrahydroxy-2-[2-(3-hydroxy-4-methoxyphenyl)ethyl]-5,6,7,8-tetrahydro-4H-chromen-4-1*, as given in Figure 5C. Consequently, this shows the stronger binding and inhibitory potential of these compounds to rescue the host immune response against the COVID-19 infection.

### 2.4. Structural Compactness Analysis

**Figure 6:**
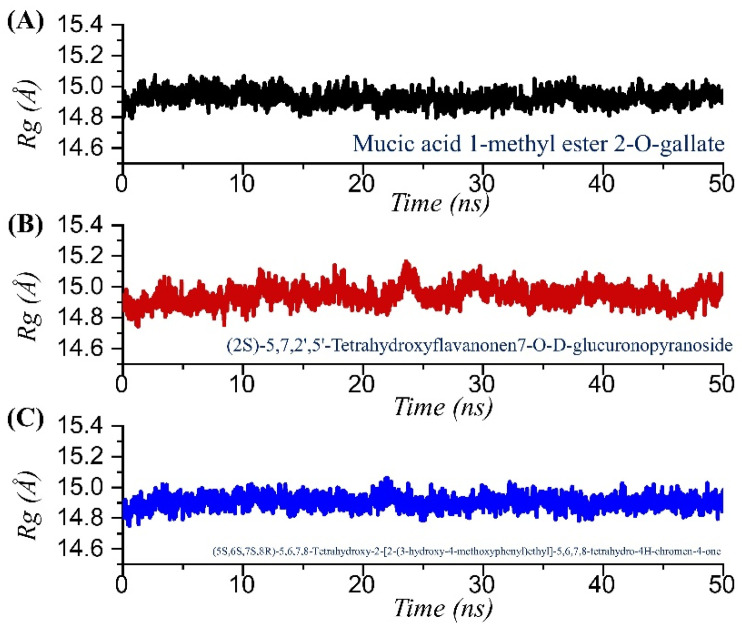
Structural compactness analysis in a dynamic environment of the TCM42798, TCM47007, and TCM30675 complexes calculated as Rg. (**A**) The Rg graph for mucic acid 1-methyl ester 2-O-gallate. (**B**) The Rg graph for TCM47007 or (2S)-5,7,2’,5’-Tetrahydroxyflavanone 7-O--D-glucuronopyranoside. (**C**) The Rg graph for TCM30675 or (5S,6S,7S,8R)-5,6,7,8-Tetrahydroxy-2-[2-(3-hydroxy-4-methoxyphenyl)ethyl]-5,6,7,8-tetrahydro-4H-chromen-4-1 complex.

Analysis of structural compactness in a dynamic environment is essential to understand the binding and unbinding events that happened during the simulation. These events demonstrated significant information regarding the binding stability and could be used to select the best compounds for the inhibition potential determination in the experimental setup. Thus, to determine the structural compactness, we calculated radius of gyration (Rg) over the simulation time as a function of time for each complex. As given in Figure 6A–C, all the complexes followed a similar pattern as RMSD. The TCM42798 complex demonstrated a uniform pattern of Rg with no increase or decrease over the simulation time. The structure reported no significant deviation, and the average Rg was reported to be 14.90 Å. The Rg for TCM42798 is shown in Figure 6A. On the other hand, TCM47007 demonstrated a small deviation between 23 and 28 ns and then reported a stable straight graph, which showed the stable binding steered by different kinds of bonds between TCM47007 and Mac-I. The TCM47007-Mac-I complex reported an average Rg of 14.96 Å. The Rg for TCM47007 is shown in Figure 6B. Furthermore, TCM30675 also reported a similar pattern of Rg as RMSD with no major significant deviation. A straight uniform Rg graph can be seen in Figure 6C, where a small fluctuation between 20 and 22 ns can be observed, while the Rg then stabilized and no significant deviation was reported. An average Rg of 15.90 Å was also reported for TCM30675. The Rg for TCM30675 is shown in Figure 6C.

### 2.5. Residues Flexibility Profiling

**Figure 7:**
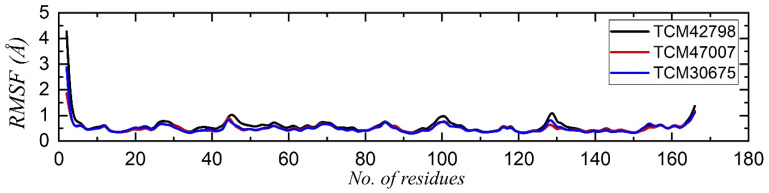
Residual flexibility analysis of the TCM42798, TCM47007, and TCM30675 complexes.

Residue flexibility is strongly correlated with the functional relevance of a protein, as it confers strength to the binding between the interacting molecules. Assessment of residue flexibility for the key residues demonstrates the impact of the small molecule binding. Thus, to determine how the residue flexibility is affected by the binding of these drugs, we calculated the root mean square fluctuation (RMSF) for each complex. As given in Figure 7, all the complexes demonstrated a similar pattern of residue flexibility. All the residues in each complex displayed minimal fluctuation, which showed the stronger binding of these compounds. The regions between 40 and 50, 90 and 100, and 120 and 140 demonstrated comparatively higher fluctuations due to the loop distribution.

### 2.6. Hydrogen Bonding Analysis

**Figure 8:**
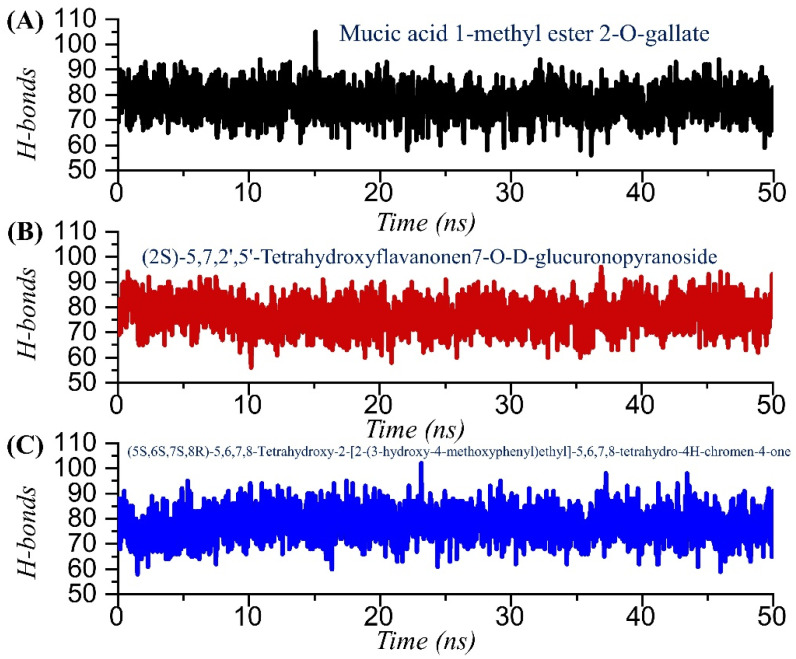
Hydrogen bonding analysis of the TCM42798, TCM47007, and TCM30675 complexes. (**A**) The H-bonds graph for mucic acid 1-methyl ester 2-O-gallate. (**B**) The H-bonds graph for TCM47007 or (2S)-5,7,2’,5’-Tetrahydroxyflavanone 7-O--D-glucuronopyranoside. (**C**) The H-bonds graph for TCM30675 or (5S,6S,7S,8R)-5,6,7,8-Tetrahydroxy-2-[2-(3-hydroxy-4-methoxyphenyl)ethyl]-5,6,7,8-tetrahydro-4H-chromen-4-1 complex.

Hydrogen bonds confer strength to the binding of small molecules. The binding of small molecules is steered by hydrogen and many other bonds, which consequently show the inhibitory features. To determine the binding strength, we calculated the total number of hydrogen bonds and the population, and the simulation trajectories were analyzed. In TCM42798 or mucic acid 1-methyl ester 2-O-gallate, the average number of hydrogen bonds was reported to be 82, while in the case of TCM47007 or (2S)-5,7,2’,5’-Tetrahydroxyflavanone 7-O--D-glucuronopyranoside, the average number of hydrogen bonds was also reported to be 79, while TCM30675 or the (5S,6S,7S,8R)-5,6,7,8-Tetrahydroxy-2-[2-(3-hydroxy-4-methoxyphenyl)ethyl]-5,6,7,8-tetrahydro-4H-chromen-4-1 complex reported 78 average hydrogen bonds. The total number of hydrogen bonds in each complex is shown in Figure 8A–C. Moreover, the hydrogen bonding population for the key interacting residues was estimated and revealed in each complex, namely Ala1060 (63%, 74%, 58%), Asn1062 (66%, 53%, 61%), Gly1068 (51%, 43%, 34%) Val1071 (32%, 24%, 23%), Ala1072 (4%, 17%, 13%), Leu1148 (1%, 6%, 12%), Ala1176 (0%, 0%, 7%), and Phe1178 (0.36%, 2%, 11%) in TCM42798, TCM47007, and TCM30675 complexes, respectively. Together these results show that these compounds bind more strongly to the Mac-I and thus produce inhibitory properties that could help to rescue the host immune response against COVID-19 infection.

### 2.7. Binding Free Energy Estimation

The binding strength of small molecules using the binding free energy method, MM-GBSA, is a widely used method for re-demonstrating docking stability and correct binding. The above-mentioned MM-GBSA technique is more computationally economical than the more expensive alchemical free energy method. When compared to rational scoring functions, it is one of the most accurate techniques. Keeping in mind the implementation of this approach, we also employed the same method to compute the binding free energy for TCM42798, TCM47007, and TCM30675 complexes. The vdWs for these complexes were reported to be −84.26 kcal/mol, −59.79 kcal/mol, and −53.24 kcal/mol, respectively, while the electrostatic energies were reported to be −12.22 kcal/mol, −13.22 kcal/mol, and −15.66 kcal/mol, respectively. Moreover, the ESURF values were reported to be 17.45 kcal/mol, 14.68 kcal/mol, and 12.25 kcal/mol, respectively. The total binding free energy for these complexes was reported to be −69.78 kcal/mol, −50.11 kcal/mol, and −47.64 kcal/mol, respectively, which consequently reflected the stronger binding and inhibitory potential of these compounds. The MM/GBSA results are given in Table 2.

## 3. Material and Methods

### 3.1. Modelling of the Macrodomain-I (Mac-I) of B.1.1.529 Variant

The experimentally reported structure in the protein database was collected through accession number 6W02 to model the V1069I mutations reported in the Mac-I of NSP3 of the omicron variant [44]. For this purpose, the amino acid sequence using P0DTD1 accession number was obtained from UniProt, European union [45]. For modelling of the variant structure, AlphaFold 2.0 Seoul National University, Seoul, South Korea, was used, which is currently the best and most accurate approach for 3D structural modelling [46]. 

### 3.2. Virtual Screening of Traditional Chinese Medicine Database

The Traditional Chinese Medicine Database (TCM) currently holds 57,000 entries of different medicinal compounds isolated from Chinese herbs. It is considered as an important medicinal repository for discovering novel treatments that are safe and effective. Thus, we screened the complete TCM database against Mac-I of the B.1.1.529 variant. For screening, we used the EasyVS (http://biosig.unimelb.edu.au/easyvs, accessed on 10 April 2022) webserver [47]. Custom parameters were used and defined, such as the active site, and the RO5 filter was enabled for the filtration and removal of molecules that violated Lipinski’s rules. Furthermore, for the top 100 hits, a second round of screening was performed using the Auto Dock Vina algorithm [48]. Finally, the top-selected compounds were used for the Induced-Fit docking (IFD) approach to remove the false-positive results. The top hits were identified based on the docking score for ADPr, taken as a control, as previously reported [22]. 

### 3.3. Molecular Dynamics Simulation (MDS)

Characterization of dynamic features to accurately portray the inhibitory potential of the top hits was studied by performing molecular dynamics simulation [49]. The top three hits were investigated by using AMBER20 simulation software by adding an optimal point charge water model and sodium ions for neutralizing the effect of any charge. Protein and drug parameterization was achieved by recruiting FF19SB and GAFF forcefields. Each system after gentle minimization was subjected to heating followed by equilibration. The production runs for 50 ns each were completed. For any long-range electrostatic interactions (10.0 Å cutoff), the PME (particle mesh Ewald algorithm) was used, while covalent bonds were treated with the SHAKE algorithm. 

### 3.4. Trajectories Analysis Using CPPTRAJ and PTRAJ

Evaluation of the simulation trajectories to forecast the dynamic stability, flexibility index, hydrogen bonding, structural compactness, and other parameters was completed by using CPPTRAJ and PTRAJ modules [50]. Root mean square deviation (RMSD) was estimated for stability, root mean square fluctuation (RMSF) to index the flexibility of each residue, radius of gyration (Rg) to foresee the compactness, and hydrogen bonding analysis to estimate the bonding population.

### 3.5. Estimation of Post-Simulation Binding Energy

The most generally utilized strategy in many related studies is to evaluate the strength of small molecule binding by employing the binding free energy (BFE) approach [51,52,53,54]. We also adopted the MMPBSA.py script to compute the binding free energy of the protein–ligand complexes by evaluating 2500 snapshots, keeping in mind the relevance of this strategy in re-ranking the binding conformations. For this purpose, the following equation was used to estimate the BFE:

“ΔGbind=ΔGcomplex−[ΔGreceptor+ΔGligand]” 

The Δ*G_bind_* represents the total binding energy, while Δ*G_receptor_*, Δ*G_ligand_*, and Δ*G_complex_* represent the binding energy of protein, drug, and complex, respectively. The following equation was used to estimate individual binding energies such as bonded (G*_bond_*), electrostatic (G*_ele_*), polar (G*_pol_*), and non-polar (G*_npol_*), which contribute to the total binding free energy.

“G=GbondGelectrostatic−Gvan der Waal−GpolarGnon−polar”

## 4. Conclusions

The present study employs molecular modelling and MD simulation approaches to target the macrodomain-I of the B.1.1.529 variant of SARS-CoV-2. Three novel compounds, namely TCM42798, TCM47007, and TCM30675, were identified as potential inhibitors of Mac-I. These agents may suppress NSP3 directly, allowing the host immune system to recuperate. The current study lays the groundwork for the development of new drugs to combat SARS-CoV-2 and its variants.

## Figures and Tables

**Table 1:** List of top 11 scoring compounds with their TCM IDs, compounds names, 3D structures, and docking scores.

TCM Database ID	Compound Names	2D Structures	Docking Scores
**TCM42798**	Mucic_acid_1-methyl_ester_2-O-gallate	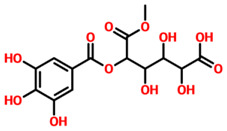	−13.70
**TCM47007**	(2S)-5,7,2’,5’-Tetrahydroxyflavanone_7-O- -D-glucuronopyranoside	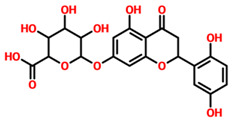	−13.25
**TCM30675**	(5S,6S,7S,8R)-5,6,7,8-Tetrahydroxy-2-[2-(3-hydroxy-4-methoxyphenyl)ethyl]-5,6,7,8-tetrahydro-4H-chromen-4-one	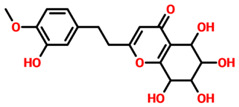	−12.49
**TCM27763**	30389	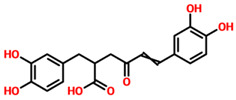	−11.93
**TCM33425**	Apigenin-bioside	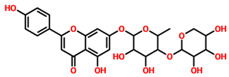	−11.72
**TCM28788**	31943	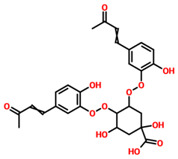	−11.46
**TCM42159**	(-)-5’-Methoxyisolariciresinol-2-O-D-xylopyranoside_(D2)	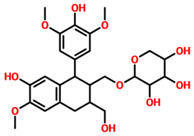	−11.45
**TCM47184**	Tibeticanol	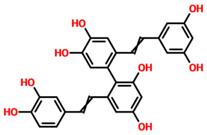	−11.36
**TCM31603**	36132	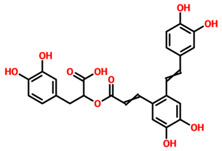	−11.04
**TCM31784**	36381	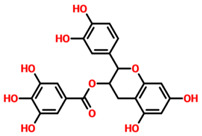	−11.02

**Table 2:** Binding free energy calculated as MM/GBSA. All the values are given in kcal/mol.

MM/GBSA	TCM42798	TCM47007	TCM30675
vdW	−84.26	−59.79	−53.24
electrostatic	−12.22	−13.22	−15.66
ESURF	17.45	14.68	12.25
EGB	9.25	8.22	9.01
∆G Bind	−69.78	−50.11	−47.64

## Data Availability

The data presented in this study are available within the article.
